# Saliva as a Diagnostic Tool for Early Detection of Exercise-Induced Oxidative Damage in Female Athletes

**DOI:** 10.3390/biomedicines12051006

**Published:** 2024-05-02

**Authors:** Aleksandr N. Ovchinnikov, Antonio Paoli

**Affiliations:** 1 Laboratory of Non-Invasive Diagnostics in Sports, Department of Sports Medicine and Psychology, Lobachevsky University, 603022 Nizhny Novgorod, Russia; 2 Department of Biomedical Sciences, University of Padua, 35122 Padua, Italy; antonio.paoli@unipd.it

**Keywords:** biomarkers, female athletes, lipid peroxidation, non-invasive diagnostics, oxidative damage, saliva, swimming

## Abstract

Although blood still remains the most commonly utilized medium to detect increased levels of oxidative damage induced by exercise, saliva diagnostics have gained increasing popularity due to their non-invasive nature and athlete-friendly collection process. Given that the contribution of various phases of the menstrual cycle to the levels of oxidative damage may differ, the aim of this study was to evaluate an agreement between salivary and plasmatic levels of lipid peroxidation products in female swimmers in both the follicular (F) and luteal (L) phases of the menstrual cycle at rest and following exercise. Twelve well-trained female swimmers aged 19.6 ± 1.1 years old were examined. We measured diene conjugates (DCs), triene conjugates (TCs), and Schiff bases (SBs) in lipids immediately after their extraction from both saliva and blood plasma. All female swimmers were studied two times each, in the two different phases of one menstrual cycle, before and after high-intensity interval exercise (HIIE). Salivary and plasmatic levels of DCs, TCs, and SBs significantly increased post-exercise compared to pre-exercise, in both the F and L phases. A high positive correlation was observed between the concentrations of DCs, TCs, and SBs in the saliva and blood plasma of participants in the F and L phases, both at rest and following HIIE. Ordinary least products regression analysis indicates that there was no proportional and differential bias in the data. The Bland–Altman method also declares that there was no differential bias, since the line of equality was within the 95% confidence interval of the mean difference between salivary and plasmatic levels of DCs, TCs, and SBs in female swimmers, in both the F and L phases, before and after HIIE. There was also no proportional bias in the Bland–Altman plots. Thus, this is the first study to report a high agreement between the quantifications of DCs, TCs, and SBs in the saliva and blood plasma of female swimmers in both the F and L phases, at rest and following HIIE.

## 1. Introduction

The presence of reactive oxygen species (ROS) can be inferred by their effects on lipids, nucleic acids, carbohydrates, and proteins, to produce specific compounds which, so long as they cannot be generated by other mechanisms, may be used as “biomarkers” of oxidative damage [1]. The early detection of any unnecessary oxidative damage in athletes is crucial for optimizing athletic performance, preventing injury, promoting recovery, and protecting long-term health [2]. Many athletes, including swimmers, often undergo high-intensity interval exercises (HIIEs), which are incorporated into their training programs to improve athletic performance. However, HIIEs may cause a considerable increase in ROS production, leading to alterations in redox milieu, as evidenced by elevated levels of certain biomarkers of oxidative damage, such as lipid peroxidation (LPO) products, in both blood plasma and saliva [3,4,5,6,7,8,9,10,11]. Although plasma still remains the most commonly utilized medium to measure biomarkers capable of identifying exercise-induced oxidative stress, in recent years, there has been growing evidence supporting the hypothesis that saliva may be considered as a more attractive option, mainly due to its safe, simple, and stress-free sampling [6,11,12]. Consequently, the development of techniques and technologies, which have a potential to use saliva as a diagnostic tool to enable more frequent, accessible, and non-invasive detection of increased levels of oxidative damage without any form of blood draw, is important.

Among the methods available for the evaluation of oxidative damage, the thiobarbituric acid-reactive substance (TBARS) assay is a widely used test, due to its advantage of being inexpensive and technically easy [5,11,13,14]. This assay aims to detect malondialdehyde (MDA), which is a split product of an endoperoxide of polyunsaturated fatty acids (PUFAs), resulting from oxidation of lipid substrates [13,14]. However, the TBARS method is nonspecific for MDA, because TBA forms chromogens from many biomolecules other than MDA [1,13,14,15,16]. The low specificity can result in a significant overestimation of MDA concentrations [1]. The use of more selective assays such as high-performance liquid chromatography (HPLC) coupled to ultraviolet (UV), fluorescence, or mass spectrometry (MS) detectors to separate the “real” TBA–MDA adduct from false chromogens increases specificity [13,17,18], but does not eliminate all problems [1]. Furthermore, MDA is not a compound produced exclusively by LPO process, which may limit its usefulness as a biomarker of oxidative lipid damage [19]. Therefore, MDA analysis alone and/or the TBA test is not able to provide accurate information on LPO and its propagation rates imposed by exercise.

Prominent among LPO products that have been measured using MS-based methods are the F_2_-isoprostanes (F_2_-IsoPs) [20]. However, it should be noted that they are one of many LPO end products and that the concentrations of their various types may be influenced by experimental conditions [1]. In addition, F_2_-IsoPs can arise from the free-radical-induced, non-enzymatic oxidation of, exclusively, arachidonic acid. Despite the fact that F_2_-IsoPs can be separated from those which are generated from the enzymatic oxidation of arachidonic acid [1], other PUFAs are also readily available for peroxidation, which is not taken into consideration when measuring F_2_-IsoPs only. Moreover, current approaches to quantify F_2_-IsoPs, in particular gas chromatography–MS and LC–tandem MS (LC–MS/MS) techniques, are not without disadvantages, which include high purchase, maintenance, and operational costs; lack of portability; as well as the infrastructure needs for maintaining the power, gases, and exhaust for each system [21]. Given that in all studies aimed to quantify one F_2_-IsoP isomer, such as 8-iso-PGF_2_α, there was poor agreement between MS-based methods and commercially available immunoassay kits [22,23,24,25], further validation is required [1].

UV absorbance for measuring diene conjugates (DCs), triene conjugates (TCs), and Schiff bases (SBs) is an alternative approach, which can accompany a more specific method, such as LC–MS/MS, to assess the whole process of lipid peroxidation. Although precise measurements of DCs, TCs, and SBs can rarely be carried out using UV spectrophotometry directly in body fluids, due to the presence of interfering UV-absorbing molecules which do not arise from LPO process, this technique is suitable for use in lipids after their extraction from the body fluids [1]. DCs are the major primary products of LPO, which are formed as a result of the rearrangement of the double bond [6,26,27,28]. They have two double bonds separated by a single bond and show a characteristic absorbance in the UV region at a wavelength of approximately 232 nm [6,26,27,29]. TCs, which absorb at around 278 nm, are the LPO products with three double bonds separated by a single bond [6,26,27]. SBs are one of the LPO end products that are derived from the reaction between the C1 carbonyl group of 4-hydroxynonenal (4-HNE) and primary amines [6,30,31]. Despite some evidence suggesting that there are slow and reversible kinetics of the SB’s formation, Schiff bases are more stable products of LPO than 4-HNE [6]. The presence of SBs in a sample is routinely determined on the basis of strong absorbance at 400 nm [6,26,27]. Thus, UV spectrophotometry capable of simultaneously detecting DCs, TCs, and SBs may be used as a comparatively rapid, technically simple, economically cheap, and fairly accurate method, allowing for the identification of increased LPO and, thereby, oxidative stress caused by exercise.

Females and males differ in oxidative stress mechanisms, as evidenced by sexual dimorphism in humans, in both the production and decomposition of ROS [32]. First, females have a higher expression and activity of key antioxidant enzymes such as superoxide dismutase and glutathione peroxidase. Furthermore, females have a lower rate of electron leakage in the mitochondrial electron transport chain and a lower activity of pro-oxidant enzymes, such as xanthine oxidase and nicotinamide adenine dinucleotide phosphate (NADPH) oxidase [32]. Since several types of muscular exercise, including HIIE, have been reported to induce similar changes in the salivary and plasmatic concentrations of LPO products in male athletes, as demonstrated by a high agreement between their measurements in the two biological fluids [6,33], further studies investigating whether salivary levels of DCs, TCs, and SBs can also represent their plasma content in female athletes under exercise conditions are important for sports medicine. Given that the contribution of various phases of the menstrual cycle to the LPO propagation rates may differ, the aim of this research was to estimate an agreement between the quantifications of DCs, TCs, and SBs in the saliva and blood plasma of female swimmers in both the follicular and luteal phases of the menstrual cycle, at rest and following HIIE.

## 2. Materials and Methods

### 2.1. Participants and Study Design

While previous power analyses [10] suggested that HIIE-induced differences in LPO products in both plasma and saliva could be detected with sample sizes as low as 3 to 9 participants (given a power of 80%, with an α of 0.05), we conservatively enrolled 12 female swimmers (age: 19.58 ± 1.08 years; height: 175.75 ± 2.34 cm; body mass: 60.75 ± 2.30 kg; body mass index: 19.66 ± 0.39 kg/m^2^), with a minimum of five years of competition experience. They had regular menstrual cycles (cycle length: 28 to 32 days) and did not take contraceptive pills for more than three months before this study. None had a history of pregnancy. All participants were systemically healthy, asymptomatic, normotensive, and non-smokers. They had no significant medical history and were free of medication. Exclusion criteria for the participants were respiratory infection, periodontal diseases, dental treatment, dental materials, ingestion of antioxidant supplements, and orthopedic injury (contusion, sprain, or fracture).

The study was approved by the Bioethics Committee of Lobachevsky University (Nr. 43) and was conducted in accordance with the guidelines laid down in the Declaration of Helsinki [34]. Each female athlete gave written informed consent prior to enrollment in this study, after thorough explanation of the study design and protocol. After signing informed consent, they were invited to the laboratories of Lobachevsky University. During a visit, all participants experienced medical screening to meet eligibility criteria. Additionally, they were instructed to control their diet and avoid taking any antioxidant supplements during this study. Participants were also instructed to have breakfast 1 h prior to exercise testing and to refrain from ingesting caffeine and alcohol for at least 24 h prior to blood and saliva sampling. Adherence to dietary habits was assessed by a qualified dietician via an interview.

The group of swimmers comprised short-distance athletes who kept a typical training routine, which was identical for all participants. Weekly training was at least  ≥5 sessions, for at least  ≥1 h per training session. Each female swimmer was studied two times in one menstrual cycle. The two different phases in one menstrual cycle are early follicular phase (1–7 days) and luteal phase. Menstrual cycles were tracked in order to determine the length of each phase accurately. This was carried out by noting the start and end dates of menstruation and observing changes in basal body temperature every morning during this study. The luteal phase was defined as 5 to 7 days after obvious elevation of morning body temperature. Exercise testing was conducted once in each phase at a similar time (±1 h) of the day. All participants did not exercise for 24 h prior to exercise testing. On the day of the exercise testing, athletes underwent HIIE, which consisted of four bouts of 50 m distance swimming at top speed, interspersed with a 45 s rest between the bouts. HIIE was performed in a 25 m indoor swimming pool in the morning. Participants were encouraged to produce a maximal effort in each of the four bouts, using their favorite stroke. The time of each bout was recorded manually using a stopwatch. The average time was calculated by dividing by four the total time taken to complete all four bouts. Because there was no stroke uniformity requirement for swimmers, to unify the participants’ results, average time was converted into the number of points according to a single assessment system, which was developed and approved by the International Swimming Federation (FINA points). HIIE type was selected due to its widespread use in the training process among the swimmers that participated in this study. Participants were examined for saliva and blood sampling before and immediately after HIIE, followed by a measurement of LPO products.

### 2.2. Blood and Saliva Sampling

Blood (4 mL) was drawn from the median basilic vein at each time and then put in tubes containing EDTA. Unstimulated saliva was collected into sterile tubes for three min using the spitting method. Before saliva sampling, swimmers thoroughly rinsed their mouth and then swallowed the remaining water. Each specimen of blood and saliva was obtained no more than fifteen min prior to the exercise testing and immediately (within fifteen min) after HIIE, in the morning. All samples were stored at 4 °C in a portable cooler, during transport to the laboratory. Saliva and blood specimens were centrifuged at 3000 rpm for fifteen min and then the supernatant was aliquoted. All aliquots were kept at −40 °C until the time of measurement of LPO products.

### 2.3. Quantification of Lipid Peroxidation Products

DCs, TCs, and SBs were measured in lipids using a spectrophotometer “SF-2000” (OKB SPECTR, Saint-Petersburg, Russia). To extract lipids from a sample, 8 mL of a heptane–isopropanol mixture in a 1:1 ratio was added to 0.5 mL of plasma or saliva, with subsequent blending for fifteen min. As is known, heptane can extract mainly neutral lipids, but phospholipids may be extracted by isopropanol [35]. In this way, it becomes possible to measure LPO products in various classes of lipids. Lipid extract was centrifuged at 3000 rpm for fifteen min. Immediately after this, 5 mL of heptane–isopropanol mixture in a 3:7 ratio was added to the lipid extract. After blending, 2 mL of aqueous solution of HCl (0.01 N) was added, in order to separate the heptane phase from the isopropanol phase, as well as to remove the non-lipid impurities. Following phase separation, the heptane (upper) phase of the lipid extract was transferred to a clean tube. To dehydrate the isopropanol (lower) phase of the lipid extract, 1 g of NaCl was added. The isopropanol phase was then transferred to a clean tube. Optical density (OD) was measured at the following wavelengths: 220 nm (absorbance of isolated double bonds), 232 nm (absorbance of DCs), 278 nm (absorbance of TCs), and 400 nm (absorbance of SBs) [6,10,27,33,35]. Each phase was assessed against the corresponding control sample that was prepared in the same manner as the test sample, but distilled water was added instead of saliva or plasma. The concentrations of DCs, TCs, and SBs in a sample were presented as continuous variables with relative units and were calculated using the following equations [6,10,33]:

(1) $$\mathrm{DC}=\left(\mathrm{OD}232\mathrm{UP}/\mathrm{OD}220\mathrm{UP}\;+\;\mathrm{OD}232\mathrm{LP}/\mathrm{OD}220\mathrm{LP}\right)\times 0.14,$$

(2) $$\mathrm{TC}=\left(\mathrm{OD}278\mathrm{UP}/\mathrm{OD}220\mathrm{UP}\;+\;\mathrm{OD}278\mathrm{LP}/\mathrm{OD}220\mathrm{LP}\right)\times 0.16,$$

(3) $$\mathrm{SB}=\left(\mathrm{OD}400\mathrm{UP}/\mathrm{OD}220\mathrm{UP}\;+\;\mathrm{OD}400\mathrm{LP}/\mathrm{OD}220\mathrm{LP}\right)\times 52,$$

where OD220UP, OD232UP, OD278UP, and OD400UP—optical density or absorbance of the upper phase of the lipid extract at 220 nm, 232 nm, 278 nm, and 400 nm, respectively; OD220LP, OD232LP, OD278LP, and OD400LP—optical density or absorbance of the lower phase of the lipid extract at 220 nm, 232 nm, 278 nm, and 400 nm, respectively; 0.14, 0.16, 52—correction coefficients.

### 2.4. Statistical Analysis

**Figure 1:**
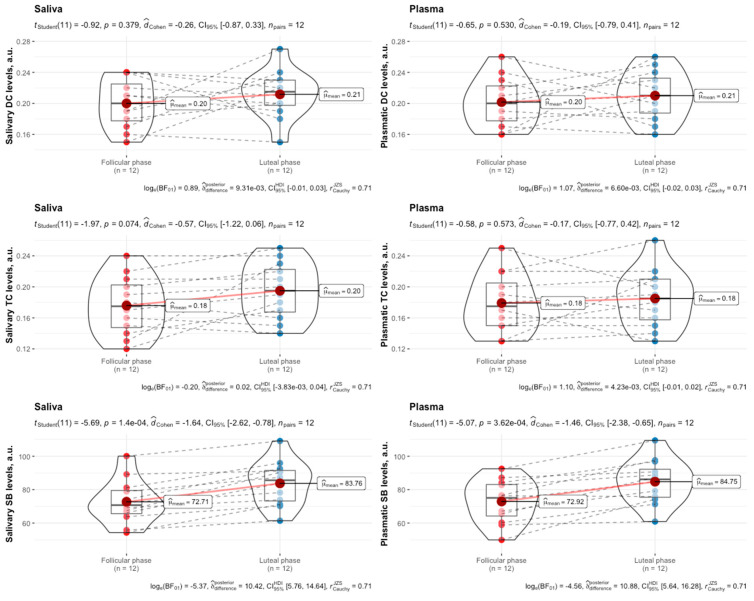
Salivary and plasmatic levels of DCs, TCs, and SBs in female swimmers in the follicular and luteal phases of the menstrual cycle at rest. Data are expressed as means, augmented with the medians and interquartile ranges, and are compared using paired-sample *t*-tests. DCs, diene conjugates; TCs, triene conjugates; SBs, Schiff bases.

**Figure 2:**
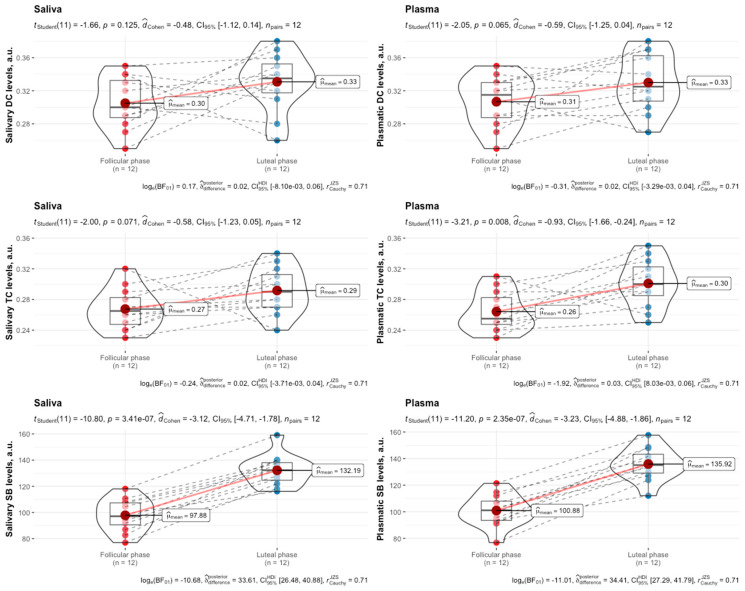
Salivary and plasmatic levels of DCs, TCs, and SBs in female swimmers in the follicular and luteal phases of the menstrual cycle following high-intensity interval exercise. Data are expressed as means, augmented with the medians and interquartile ranges, and are compared using paired-sample *t*-tests. DCs, diene conjugates; TCs, triene conjugates; SBs, Schiff bases.

**Figure 3:**
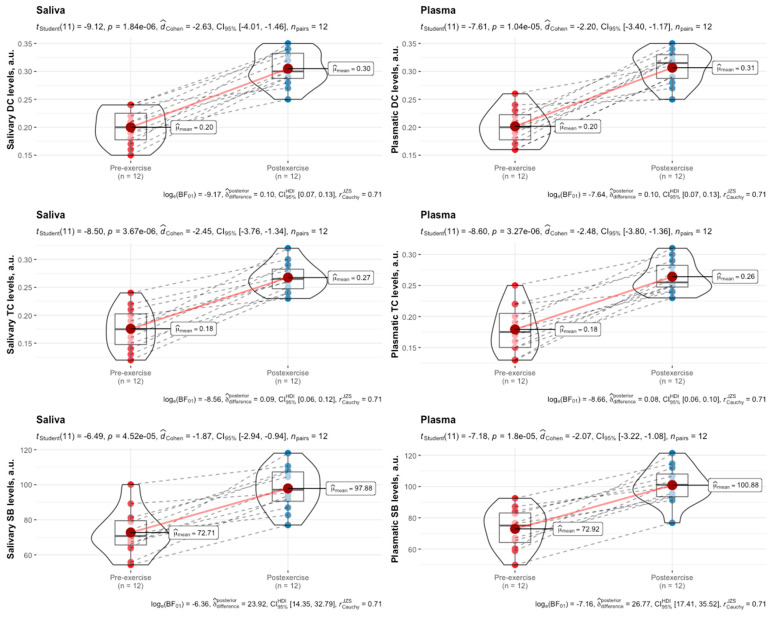
Salivary and plasmatic levels of DCs, TCs, and SBs in female swimmers in the follicular phase of the menstrual cycle before and immediately after high-intensity interval exercise. Data are expressed as means, augmented with the medians and interquartile ranges, and are compared using paired-sample *t*-tests. DCs, diene conjugates; TCs, triene conjugates; SBs, Schiff bases.

**Figure 4:**
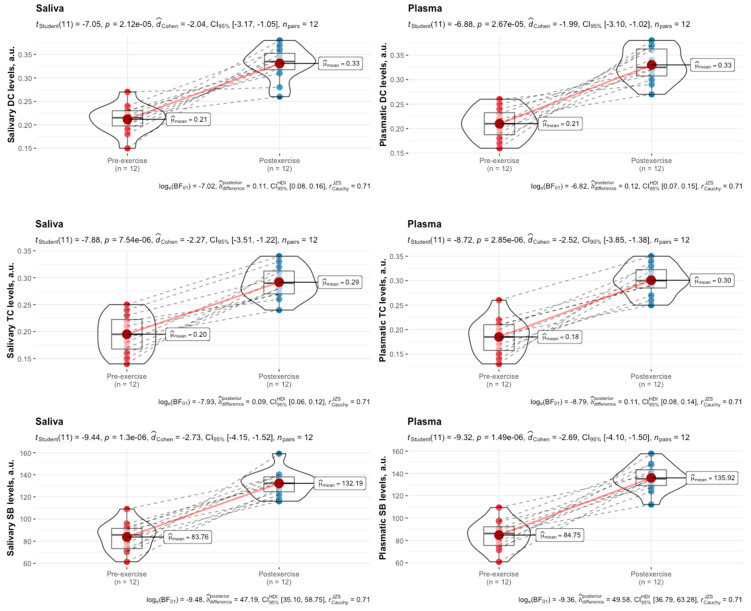
Salivary and plasmatic levels of DCs, TCs, and SBs in female swimmers in the luteal phase of the menstrual cycle before and immediately after high-intensity interval exercise. Data are expressed as means, augmented with the medians and interquartile ranges, and are compared using paired-sample *t*-tests. DCs, diene conjugates; TCs, triene conjugates; SBs, Schiff bases.

Data in the text and Table 1 are reported as mean ± standard deviation (SD). Data in Figure 1, Figure 2, Figure 3 and Figure 4 are expressed as the mean, augmented with the median and interquartile range. The assumption of normality was estimated by using a Shapiro–Wilk test. Given that all data were normally distributed, within-group differences were examined using paired-sample *t*-tests, augmented with the calculation of respective effect sizes. Considering the sample size and given a power of 80% with an α of 0.05, an effect size of Cohen’s *d*  =  0.89 would be needed to determine within-group differences in the levels of the LPO products. Pearson’s correlation coefficient was used to indicate significant linear relationship among levels of the same LPO products in plasma and saliva and the ordinary least squares (OLS) regression line was displayed. Ordinary least products (OLP) regression analysis and the Bland–Altman method of differences were applied to determine both a differential bias (i.e., a constant difference between salivary and plasmatic levels of the LPO product) and a proportional bias (i.e., a difference which depends on the value of the mean of salivary and plasmatic levels of the LPO product). Since the differences between salivary and plasmatic levels of the same LPO products were normally distributed, assumptions of the Bland–Altman method were met. A value of *p* < 0.05 was considered as significant. Bonferroni correction was applied, if required. All statistical tests were performed using RStudio software, version 2022.07.2+576 for macOS (RStudio, PBC, Boston, MA; http://www.rstudio.com).

## 3. Results

All participants tolerated the studies well. Table 1 shows the HIIE performance of female swimmers in the two different phases of one menstrual cycle.

There was no significant difference (*p* = 0.64) in exercise performance between the follicular and luteal phases of the menstrual cycle in female swimmers. Although the purpose of this study was not to determine whether differences in the levels of LPO products between the follicular and luteal phases of one menstrual cycle in female swimmers exist, the outcomes are given in Figure 1 and Figure 2.

The levels of the LPO products, especially SB content, in both plasma and saliva were lower in the follicular phase compared to the luteal phase of the menstrual cycle in female swimmers before and after HIIE.

Salivary and plasmatic levels of DCs, TCs, and SBs significantly increased post-exercise compared to pre-exercise in female swimmers in the follicular phase of the menstrual cycle (Figure 3).

In the luteal phase, concentrations of DCs, TCs, and SBs were also elevated in response to HIIE in both saliva and plasma (Figure 4).

**Figure 5:**
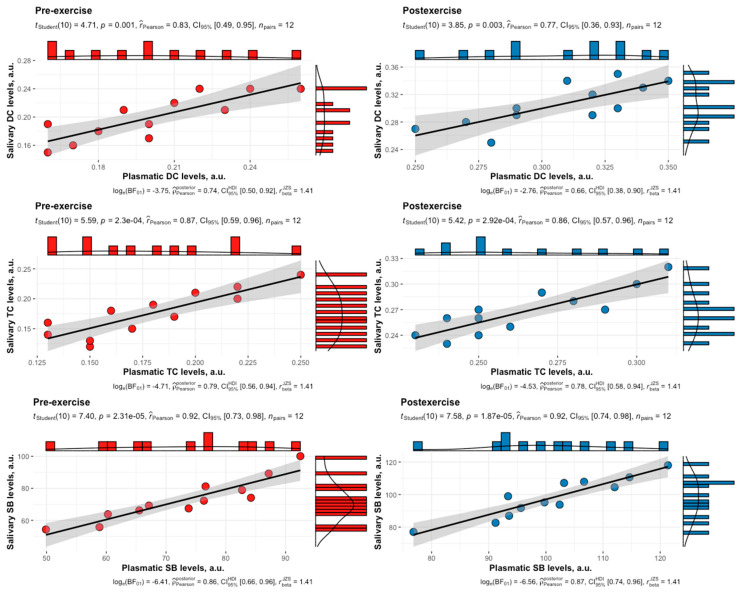
Correlation between plasmatic and salivary levels of DCs, TCs, and SBs in female swimmers in the follicular phase of the menstrual cycle at both pre-exercise and post-exercise. The solid black lines represent the ordinary least squares regression of salivary levels on plasmatic levels. The grey shaded regions indicate the 95% confidence intervals. DCs, diene conjugates; TCs, triene conjugates; SBs, Schiff bases.

A high positive correlation was observed between plasmatic and salivary levels of DCs, TCs, and SBs in female swimmers in the follicular phase of the menstrual cycle both at rest and following HIIE (Figure 5).

**Figure 6:**
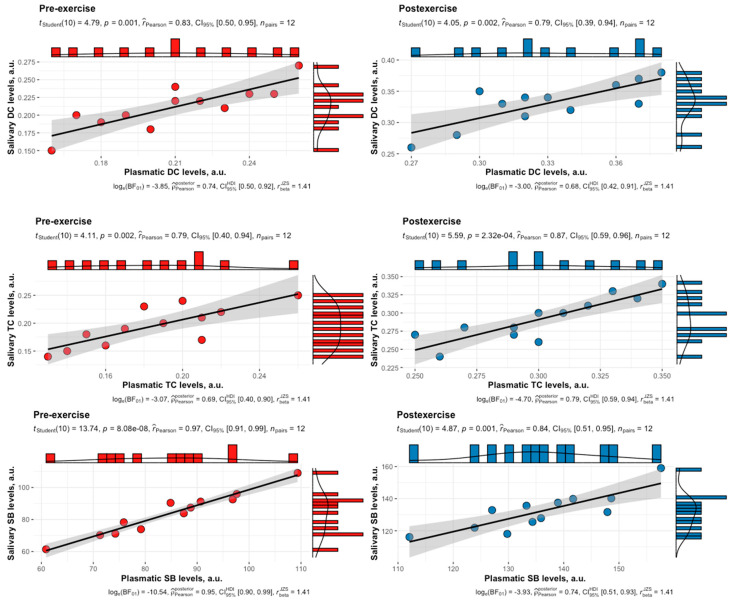
Correlation between plasmatic and salivary levels of DCs, TCs, and SBs in female swimmers in the luteal phase of the menstrual cycle at both pre-exercise and post-exercise. The solid black lines represent the ordinary least squares regression of salivary levels on plasmatic levels. The grey shaded regions indicate the 95% confidence intervals. DCs, diene conjugates; TCs, triene conjugates; SBs, Schiff bases.

A strong positive correlation was also verified between plasmatic and salivary levels of the same LPO products that were measured in female swimmers in the luteal phase, both before and after HIIE (Figure 6).

OLP regression analysis indicates that there was no proportional or differential bias in the data, i.e., the 95% confidence interval (CI) for the slope included 1 and the 95% CI for the intercept included 0, respectively (Table 2).

**Figure 7:**
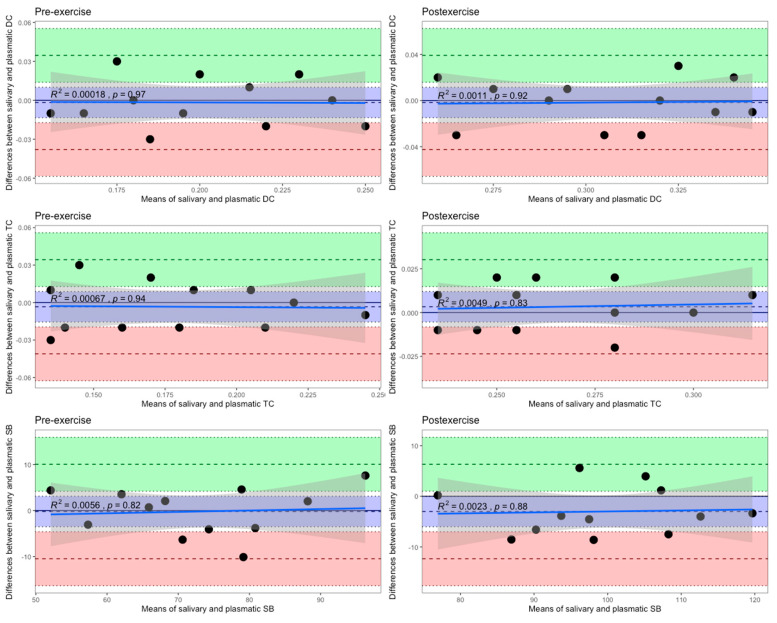
Bland–Altman plots of differences against means for comparisons of salivary and plasmatic levels of the LPO products measured in female swimmers in the follicular phase of the menstrual cycle both pre-exercise and post-exercise. The mean difference is represented by a dashed line (inside the blue area) parallel to the *x* axis. The limits of agreement are represented by dashed lines parallel to the *x* axis at −1.96 SD (inside the red area) and +1.96 SD (inside the green area). Shaded areas represent the 95% confidence interval limits for the mean difference (blue shading) and for the agreement limits (red and green shading). The solid blue lines represent the ordinary least squares regression of differences on means. The grey shaded regions indicate the 95% confidence intervals. DC, diene conjugate; TC, triene conjugate; SB, Schiff base.

**Figure 8:**
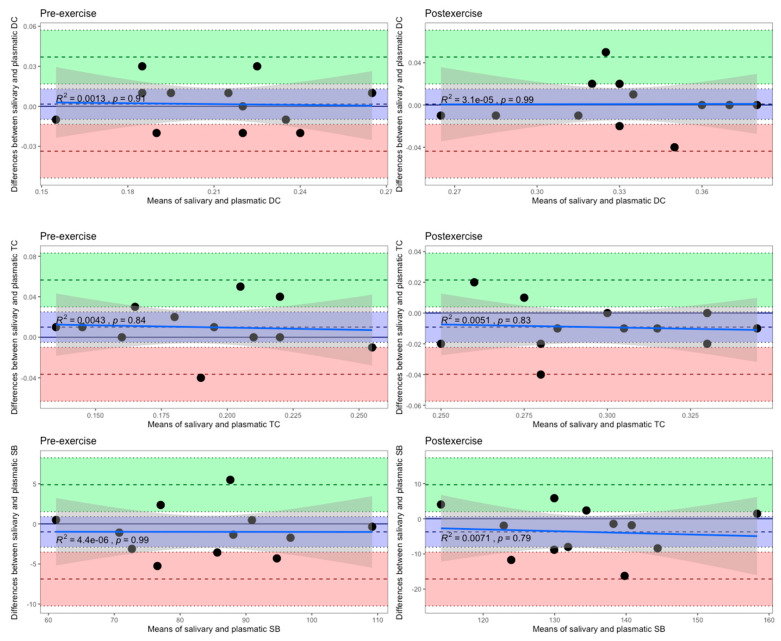
Bland–Altman plots of differences against means for comparisons of salivary and plasmatic levels of the LPO products measured in female swimmers in the luteal phase of the menstrual cycle both pre-exercise and post-exercise. The mean difference is represented by a dashed line (inside the blue area) parallel to the *x* axis. The limits of agreement are represented by dashed lines parallel to the *x* axis at −1.96 SD (inside the red area) and +1.96 SD (inside the green area). Shaded areas represent the 95% confidence interval limits for the mean difference (blue shading) and for the agreement limits (red and green shading). The solid blue lines represent the ordinary least squares regression of differences on means. The grey shaded regions indicate the 95% confidence intervals. DC, diene conjugate; TC, triene conjugate; SB, Schiff base.

The Bland–Altman method of differences also declares that there was not any significant differential bias, since the line of equality was within the 95% confidence interval of the mean difference between the salivary and plasmatic levels of each LPO product, which was measured in female swimmers in both the follicular (Figure 7) and luteal (Figure 8) phases of one menstrual cycle at rest and following HIIE.

Furthermore, there was also no proportional bias in the Bland–Altman plots, because the slope of the OLS regression of differences on means did not differ significantly from 0 (*p* > 0.05) and, equivalently, the 95% CI for the slope included 0. In this case, the limits of agreement were constructed without first finding a suitable transformation of the data. All data points were observed inside ±1.96 SD of the mean difference.

## 4. Discussion

This is the first study to report a high agreement between the quantifications of DCs, TCs, and SBs in the saliva and blood plasma of female athletes at rest and following exercise. Furthermore, similar levels of the same LPO products in plasma and saliva, both before and immediately after HIIE, were detected in all participants in the two different phases of one menstrual cycle. Although the exact mechanisms behind the movement of LPO products between blood plasma and saliva are not yet fully understood, there are multiple factors which may contribute to this phenomenon. First, LPO products can passively diffuse from blood to saliva or vice versa, due to their relatively small size and lipid solubility [6]. Second, certain transporters present on the plasma membranes of salivary gland cells and/or blood vessel cells can actively transport LPO products between blood and saliva. Third, LPO products can also move between blood and saliva, via the gaps between cells (paracellular transport) in the salivary glands and blood vessels [6,36,37,38,39]. Therefore, the salivary content of DCs, TCs, and SBs may reflect their blood concentrations.

The exact origin of ROS in blood plasma and saliva at rest is largely unknown. Blood interacts with all organs and tissues and, thus, with many possible sources of ROS. Furthermore, blood cells and plasma can autonomously generate a large number of ROS [40]. However, it should be noted that ROS could be produced as a result of normal metabolic processes in the body, depending on the phase of the menstrual cycle, given that all participants were systemically healthy and had no respiratory infection and/or periodontal disease, which can be responsible for increased ROS production in oral tissues, gums, and saliva [41,42]. Moreover, some possible exogenous sources of ROS in the oral cavity, such as xenobiotics (ethanol, cigarette smoke, and drugs), food (high-fat diet and caffeinated beverages), dental treatment (surgery, laser light, ultraviolet light, etc.), and dental materials (adhesive, composite resin, etc.) [41,43,44], were also excluded in this study.

The results of this study demonstrate that the levels of SBs in both plasma and saliva differed significantly between the two different phases of one menstrual cycle in female swimmers before and after HIIE. The pattern of changes was visible, in spite of the background of high individual variability of SB concentrations. Hormonal effects on the levels of oxidative damage have been convincingly documented. Rising levels of progesterone during the luteal phase can lead to an increase in ROS generation, causing oxidative damage to lipids and other biological macromolecules when ROS are not properly neutralized by antioxidants [45]. However, it is important to note that individual differences (including individual response to training routine), environmental factors, and lifestyle can all affect the levels of oxidative damage in different phases of the menstrual cycle and may contribute to the variability of the results [45].

Exercise is one of the factors that can induce increased ROS production. In fact, ROS are intimately involved in redox signaling, thus influencing skeletal muscle adaptation to exercise training [2]. But, in response to strenuous exercise, ROS can also lead to increased levels of oxidative damage, especially when the body’s antioxidant system is not able to properly manage ROS production [2,46,47,48,49,50]. High-intensity interval swimming caused an increase in the levels of LPO products in both the plasma and saliva of female swimmers in the follicular and luteal phases of the menstrual cycle. Although oxidants could be generated in a variety of tissues during HIIE, there is a general consensus that ROS are produced predominantly by contracting skeletal muscles during exercise [2,46,48,49,50]. Possible sources of HIIE-induced ROS generation in muscle fibers include electron transport chain, phospholipase A2, and NADPH oxidases (NOXs), among which NOX2 and NOX4 are currently considered as a major contributor to the formation of the superoxide anion (O_2_^•−^), a primary member of ROS, in striated muscle during exercise [2,46,47,48,49,50]. Activation of xanthine oxidase via ischemia/reperfusion may also be implicated in O_2_^•−^ production during HIIE [47,49,50]. Erythrocytes could be the major ROS generators located in blood (due to their quantity), but their relative contribution to the ROS production during exercise is an order of magnitude less than the skeletal muscles [2,40]. On the other hand, the main contribution of white blood cells to the ROS formation is observed with a delay after exercise (rather than during or immediately after exercise), due to phagocyte infiltration caused by the ROS generated in striated muscle during exercise. The phagocytes infiltrating into muscle tissues produce additional ROS through NOX and myeloperoxidase, thus causing oxidative damage and further accelerating the inflammatory cascade into skeletal muscles after exercise, which amplifies inflammation [51].

Regardless of the site of the production, O_2_^•−^ is not able to directly initiate LPO, but can be enzymatically or spontaneously dismutated and, thus, serves as the main cellular source of secondary ROS, such as hydrogen peroxide (H_2_O_2_) [13,50]. H_2_O_2_ is a non-radical ROS with a long half-life, allowing it to diffuse both within a cell and across the cell membranes, but its derivate, the hydroxyl radical (HO^•^), is the most powerful initiator of LPO [31,46,49,50]. In the initiation phase, pro-oxidants, such as HO^•^, abstract the bis-allylic hydrogen of PUFAs, thereby generating a carbon-centered lipid radical that tends to be stabilized by a molecular rearrangement of the double bond, to form conjugated dienes or trienes [13,28,52]. The latter depends on the number of double bonds in PUFAs, which react with radical initiators. In the propagation step, the lipid radical reacts with molecular oxygen, forming a lipid peroxyl radical that interacts with an adjacent PUFA to yield a lipid hydroperoxide (LOOH) and a new lipid radical [13,28,31]. Thus, one HO^•^ can produce a large number of LOOH, until the lipid chain autoxidation is terminated, for example, by a chain-breaking antioxidant [31].

The LPO cascade forms a wide variety of oxidation products, including DCs, TCs, and SBs. DCs are regarded as LPO primary products, which refer to two double bonds separated by a single bond [6,26,27,28,29]. Since DCs can cross membranes directly, this may lead to the covalent modification of proteins far from the site of DC formation [52]. TCs may be considered as secondary products of LPO, which have three double bonds separated by a single bond in a molecule [6,26,27]. Among the end products generated during LPO, SBs can be produced through reactions of the addition of the aldehydic group to an amino group, in particular by reactions of α,β-unsaturated aldehydes, such as 4-HNE, with Lys protein residues and with lipids containing amino groups (e.g., phosphatidylethanolamine) [13,53,54]. 4-HNE-protein adducts are now accepted as being involved in the damaging action of oxidative stress. SBs, as one such adduct, which demonstrate a characteristic absorbance in the UV region at around 400 nm, were identified [6,26,27]. High levels or formation rates of SBs might indicate increased LPO and, thereby, oxidative damage, suggesting an impairment of membrane functions [55]. Importantly, cell membrane modifications triggered by LPO often precede irreversible biomolecular damage, being an early cause of cell death [10,50,56,57]. Hence, measurement of SBs, together with primary and secondary products of LPO in saliva may be useful in the daily practice of sport medicine centers, to gain information on a shift in oxidative lipid damage (for example, toward a greater production of terminal products), because quantification of only a single oxidation product in no way reflects the whole process of lipid peroxidation [1,13]. Consequently, the proposed non-invasive technique utilizing saliva may be envisioned to allow team physicians to detect any unnecessary exercise-induced oxidative damage in real time, as well as to further evaluate the effectiveness of different recovery methods aimed to decrease this damage in female athletes.

This study has at least one obvious limitation that derives from the origin of the sample and, therefore, should be noted. Given that all participants were well-trained female swimmers who are systemically healthy and non-smokers, as well as having no periodontal disease, the results of this study should be interpreted with caution and cannot easily be generalized to all women participating in various sports, without paying attention to the state of health and the smoking habits.

## 5. Conclusions

This is the first study that demonstrates high agreement between the measurements of DCs, TCs, and SBs in the blood plasma and saliva of female swimmers in both the follicular and luteal phases of the menstrual cycle, before and immediately after HIIE. Consequently, saliva has the ability to provide a credible assessment of oxidative damage without the need of blood sampling in female athletes. Furthermore, the proposed assay of DCs, TCs, and SBs in saliva offers novel possibilities for the development of portable analyzers or even biosensors, to identify increased levels of oxidative damage in a continuous, real-time, and nonintrusive manner, including under various exercise conditions.

## Figures and Tables

**Table 1:** High-intensity interval exercise performance of female swimmers in the follicular and luteal phases of one menstrual cycle.

	Follicular Phase	Luteal Phase
FINA points, a.u.	452.67 ± 31.85	454.25 ± 28.83

Values are mean ± SD.

**Table 2:** Outcome of analysis by ordinary least products regression.

LPO Product	Phase	Time Point	*r*	*a* [95% CI]	*b* [95% CI]
DC	F	Pre	0.83	−0.0002 [−0.08, 0.08]	0.99 [0.68, 1.46]
DC	L	Pre	0.83	0.006 [−0.07, 0.09]	0.98 [0.67, 1.43]
DC	F	Post	0.77	−0.008 [−0.15, 0.13]	1.02 [0.66, 1.57]
DC	L	Post	0.79	−0.0003 [−0.14, 0.14]	1.00 [0.66, 1.53]
TC	F	Pre	0.87	−0.001 [−0.06, 0.06]	0.99 [0.70, 1.39]
TC	L	Pre	0.79	0.02 [−0.06, 0.10]	0.96 [0.63, 1.46]
TC	F	Post	0.86	−0.006 [−0.10, 0.09]	1.04 [0.73, 1.47]
TC	L	Post	0.87	0.001 [−0.10, 0.10]	0.97 [0.69, 1.36]
SB	F	Pre	0.92	−2.38 [−23.48, 18.71]	1.03 [0.78, 1.35]
SB	L	Pre	0.97	−0.96 [−14.49, 12.58]	1.00 [0.85, 1.17]
SB	F	Post	0.92	−4.91 [−32.97, 23.15]	1.02 [0.78, 1.33]
SB	L	Post	0.84	2.41 [−47.61, 52.43]	0.95 [0.66, 1.39]

DC, diene conjugate; TC, triene conjugate; SB, Schiff base. F, follicular phase; L, luteal phase. Pre, pre-exercise; Post, post-exercise. *r*, Pearson’s correlation coefficient. *a*, *b*, coefficients in ordinary least products regression model E(salivary level of the LPO product) = *a* + *b*(plasmatic level of the LPO product); *a*, intercept; *b*, slope; 95% CI, 95% confidence interval.

## Data Availability

The datasets used and/or analyzed during the current study are available from the corresponding author on reasonable request.
